# Cardiac disease and depression; a direct association?

**DOI:** 10.1007/s12471-016-0868-9

**Published:** 2016-07-26

**Authors:** E. E. van der Wall

**Affiliations:** Netherlands Society of Cardiology/Holland Heart House, Utrecht, The Netherlands

At the April meeting of EuroHeartCare 2016 (Athens, Greece), Dr. Barbro Kjellström (Karolinska Institute Stockholm, Sweden) showed that patients with a sustained myocardial infarction (MI) are more depressed but are less often prescribed antidepressants than people without a sustained MI. EuroHeartCare is the annual congress of the Council on Cardiovascular Nursing and Allied Professions (CCNAP) of the European Society of Cardiology (ESC). The data presented at EuroHeartCare were derived from the Swedish PAROKRANK study showing that periodontitis increased the risk of having a first MI by 30 % [1, 2]. The study included 805 patients under 75 years (average 62 years, 81 % men) who had experienced a first MI. Symptoms of depression were found in 14 % of patients compared with only 7 % of 805 matched controls, and these symptoms were associated with a doubled risk for an MI.

A novel and intriguing finding was that MI patients less often received treatment for depression: only 16 % of the MI patients with depression received antidepressants compared with 42 % of controls with depression. These results suggest that MI patients are undertreated with antidepressants, possibly because they did not seek help for their depression, or if they did, their symptoms were not accurately recognised and managed. According to the presenters of the study, clinicians should question MI patients more explicitly about their mental well-being and carefully listen to their reply.

In the present issue of the Netherlands Heart Journal two articles pay attention to the association between different manifestations of cardiac disease and stress-related disorders such as depression, exhaustion and anxiety [3, 4]. In patients with coronary artery disease (CAD) the prevalence of depression ranges from 25–50 % and depression is considered to be an important risk factor for hypertension, MI, heart failure, and increased mortality [5–8]. From the recent MINDMAPS study (involvement of 16 countries, including the University of Groningen) it was concluded that the prevalence of depression post-MI was higher in women than in men, but that the association between depression and cardiac prognosis was worse for men. Left ventricular function was associated with depression in men only and accounted for the increased risk of all-cause mortality in depressed men versus women, suggesting that depression in men post-MI may, in part, reflect cardiovascular disease severity [9].

Smeijers et al. [3] from Tilburg University investigated whether patients with takotsubo cardiomyopathy had increased levels of psychological distress (depressive symptoms, perceived stress, general anxiety), illness-related anxiety and personality factors. Takotsubo cardiomyopathy is a transient condition characterised by severe left ventricular dysfunction combined with symptoms and signs mimicking acute MI, predominantly occurring in women [10–13]. The authors studied 18 patients with takotsubo cardiomyopathy (mean age 68.3 ± 11.7 years, 77.8 % women) and two comparison groups: 19 healthy controls (60.0 ± 7.6 years, 68.4 % women), and 19 patients with chronic heart failure (68.8 ± 10.1 years, 68.4 % women). It was shown that takotsubo cardiomyopathy was associated with higher levels of depressive symptoms, more illness-related anxiety and less openness compared with healthy controls. No differences between takotsubo cardiomyopathy and heart failure patients were found regarding the psychological measures. These data suggest that takotsubo cardiomyopathy is associated with adverse psychological factors that may persist well after the acute episode.

AL-Qezweny et al. [4] from Erasmus MC, Rotterdam studied 534 patients following percutaneous coronary intervention (PCI) of whom 135 (25 %, mean age 59 years, 71 % male) suffered from distressed personality (type D). Generally, individuals with type D personality show negative affectivity and social inhibition to be associated with depression and anxiety [14]. The primary aim of the study was to investigate the association between type D personality at 6 months post-PCI (baseline) and depression at 10 years follow-up. At baseline, the prevalence of type D personality was 25 % (135/534). Patients with type D personality were more often depressed (42 %) than those with a non-type D personality (9 %). At 10-year follow-up, 31 % of type D personality patients were depressed versus 13 % of non-type D personality patients. Consequently, PCI patients with type D personality had a 3.69 -fold increased risk for depression and a 2.72-fold increased risk for anxiety at 10 years of follow-up. Of note, the authors did not explicitly separate their findings to male or female sex in their PCI group; according to a previous study from the same group this distinction might be of significance [15]. It was concluded that the clinical use of type D personality is very important in identifying PCI patients with a high risk for depression and anxiety; this association holds for even 10 years after the PCI procedure.

To summarise, the above-mentioned studies show a direct association between a variety of cardiac diseases and stress-related disorders such as depression, exhaustion and anxiety. This was corroborated by a recent study from the University College London, which examined depression status as a risk factor for 12 cardiovascular diseases in almost 2 million men and women; it was shown that depression was prospectively associated with cardiac, cerebrovascular, and peripheral diseases, with no evidence of disease specificity [16]. Further research is therefore needed in understanding the specific pathophysiology of heart and vascular disease triggered by depression in healthy populations. As clinicians we should remain aware of the fact that many of our patients suffer from depression and that they should be adequately managed, transcending the use of cardiac medication and justifying the prescription of antidepressants [17, 18].
